# Role modelling of clinical tutors: a focus group study among medical students

**DOI:** 10.1186/s12909-015-0303-8

**Published:** 2015-02-14

**Authors:** Annette Burgess, Kerry Goulston, Kim Oates

**Affiliations:** 1 Sydney Medical School - Central, The University of Sydney, Building 63, level 4, Royal Prince Alfred Hospital, Missenden Road, Camperdown, NSW 2050 Australia; 2 Sydney Medical School, The University of Sydney, Sydney, NSW Australia

**Keywords:** Role modelling, Medical students, Clinical tutors

## Abstract

**Background:**

Role modelling by clinicians assists in development of medical students’ professional competencies, values and attitudes. Three core characteristics of a positive role model include 1) clinical attributes, 2) teaching skills, and 3) personal qualities. This study was designed to explore medical students’ perceptions of their bedside clinical tutors as role models during the first year of a medical program.

**Methods:**

The study was conducted with one cohort (n = 301) of students who had completed Year 1 of the Sydney Medical Program in 2013. A total of nine focus groups (n = 59) were conducted with medical students following completion of Year 1. Data were transcribed verbatim. Thematic analysis was used to code and categorise data into themes.

**Results:**

Students identified both positive and negative characteristics and behaviour displayed by their clinical tutors. Characteristics and behaviour that students would like to emulate as medical practitioners in the future included:

1) Clinical attributes: a good knowledge base; articulate history taking skills; the ability to explain and demonstrate skills at the appropriate level for students; and empathy, respect and genuine compassion for patients.

2) Teaching skills: development of a rapport with students; provision of time towards the growth of students academically and professionally; provision of a positive learning environment; an understanding of the student curriculum and assessment requirements; immediate and useful feedback; and provision of patient interaction.

3) Personal qualities: respectful interprofessional staff interactions; preparedness for tutorials; demonstration of a passion for teaching; and demonstration of a passion for their career choice.

**Conclusion:**

Excellence in role modelling entails demonstration of excellent clinical care, teaching skills and personal characteristics. Our findings reinforce the important function of clinical bedside tutors as role models, which has implications for faculty development and recruitment.

## Background

Role modelling has been described within medical education as the process in which “*Faculty members demonstrate clinical skills, model and articulate expertise thought processes and manifest positive professional characteristics”* [1]. There are three interrelated learning environments where role modelling takes place, including the formal, informal and hidden curriculum [2]. Through role models, medical students develop their professional competencies, values and attitudes [3]. The characteristics of a positive role model can be separated into three core areas: 1) clinical attributes; 2) teaching skills; and 3) personal qualities [4-6].

### Clinical attributes

In order to be regarded as a role model by students, an outstanding level of clinical knowledge and skills, as well as a patient centred approach are necessary [4,6-12]. Humanistic behaviours, which encompass personal attributes such as empathy, respect and compassion are a predominant theme of importance [5,6,8,13-15]. Write and Caresse (2002) state that it is important that clinical tutors take on a “role modelling consciousness” when interacting with patients in the presence of students [9].

### Teaching skills

The importance of establishing a rapport with students, creating a supportive and positive learning environment, development of particular teaching methods, and being committed to the academic and professional development of students has been identified in previous studies [8,9,12]. The amount of teaching responsibilities that one takes on has been closely associated with excellence in role modelling [7]. Additionally, the amount of patient interactions provided by the tutor within the clinical setting has been identified as important in role modelling [5,8,13].

### Personal qualities

Excellent role models should have effective interpersonal skills; a positive outlook; leadership skills; integrity and a commitment to excellence [16,17]. Dedication, honesty, politeness and enthusiasm have also been identified as important personal qualities [5], as well as being able to inspire students [14].

It is widely accepted that a large part of learning within medicine is the aspect of social practice [18]. Learning is not just about the acquisition of clinical knowledge or skills, but also facilitating student participation in medical practices [18]. Medical education entails a process of socialisation into a profession. It is important that students develop a sense of identity of their current and future roles in medicine [17]. A role model teaches primarily by example, helping to shape the professional identity of medical students in preparation for their entry into the workplace as doctors. Through clinical experience, students are encouraged to reflect on their progress towards their professional roles and responsibilities [19].

A large part of providing students with early clinical experiences is to teach them about the role of health professionals [19]. Providing experience in early years is a critical time to develop students’ value systems, an awareness of their future professional responsibilities, and to encourage humility [19]. It also offers a key time to highlight the importance of communication skills, and display the balance between social and professional skills in communication with patients [19]. Previously, the first two years of medical education have predominantly consisted of students being taught biomedical science, with very little clinical exposure. The recent global trend towards “vertically integrated” medical programs, with early clinical exposure is in part related to the growing emphasis on personal and professional development [19]. While this early exposure has been introduced, it is a good time to pause to investigate the qualities of role models that junior students admire. Doing so helps to redefine the task of medical educators in the 21st century.

Although widely reported, the importance of role modelling remains unclear when compared to more formal approaches to learning [20]. Staff can exemplify positive or negative role models and might be more or less sensitive to the needs of students and their potential and actual impact on students should not be forgotten.

The aim of this study was to investigate the way medical students perceive and have been influenced by role models they encountered as their first year clinical tutors. The three core areas of a positive role model: 1) clinical attributes; 2) teaching skills; and 3) personal qualities [4-6] offered a valuable framework to explore our aim.

## Methods

### Context

The University of Sydney offers a four year, graduate entry medical program. First year medical students enrolled in the Sydney Medical Program (SMP) attend University main campus four days per week. One day per week, students attend their “home clinical school”. Home clinical schools are based at one of six metropolitan teaching hospitals, located within a 100 km radius of main campus. There, on a weekly basis, students take part in small group, bedside clinical tutorials. Students attend 1.5 hours of physical examination teaching, and 1.5 hours of communications teaching. Physical examination teaching involves a bedside tutorial with the emphasis on physical examination skills. Communications teaching involves a bedside tutorial with the emphasis on history taking skills. Groups consist of 6 to 7 students. Each group has one **clinical tutor,** who is either a **senior specialist (consultant**), or a **registrar (house staff),** or a **general practitioner**.

The study was conducted with one cohort (n = 301) of SMP students who had completed Year 1 of the medical program in 2013.

At the commencement of Year 2 in 2014, a convenience sample (n = 100) of students were invited by email to attend one of nine focus groups. A convenience sample was used because of timetable restrictions and subsequent difficulty in gaining access to the students by researchers. The 100 students invited to attend focus groups were present at the University campus and accessible to the researchers on one particular day and time frame during the week, over a three week period. All students who chose to attend the focus groups (n = 59) provided written informed consent for participation in the study. The focus group questions specifically focused on the perceived experiences of students during their hospital based clinical tutorials.

### Data analysis

Focus group data were transcribed verbatim with each participant being assigned an anonymous identifier (1 to 59). A thematic analysis was undertaken using Framework Analysis [21]. The initial analysis was inductive and grounded in the data and conducted by the first author on a sample of the data [22]. The aim was to identify recurrent themes and subthemes in the dataset and inform the development of a coding framework [21]. All authors met to discuss and develop this coding framework after independently reading the same data sample. Coding focused on the experiences, interactions, and beliefs that influenced the students. The coding framework that was developed was used by the first author to code the entire data set. NVivo qualitative data analysis software was used for data analysis and management.

Ethics approval was obtained from The University of Sydney Human Research Ethics Committee.

## Results

A total of nine focus groups were held, with 59/100 (59%) of students voluntarily attending. Participants included 31 females and 28 males from six metropolitan clinical schools, based at Royal Prince Alfred (n = 18), Northern (n = 13), Concord (n = 10), SAN (n = 7), Westmead (n = 7), and Nepean (n = 4) hospitals.

The results are presented using the conceptual themes of clinical attributes, teaching skills and personal qualities to illustrate students’ perceptions of role modelling.

### Clinical attributes

Students were impressed by tutors’ knowledge within their area of expertise when being taught by a senior specialist (consultant), however, it was equally important that their tutors had **a good general medicine knowledge base**. It has been previously shown that students value a tutor’s generalist skills, rather than their specialist skills [23].

*“I had a neurologist but he was knowledgeable in everything, not just neurology. He’s got the balance right”. (S15)*

Students’ impressions of their tutors’ ability to impart knowledge were driven by their concerns with following the curriculum and their desire to perform well in the summative examinations. The social and cognitive congruence that registrars (house staff) offer [24] may help to explain students’ preference for registrar teaching in terms of preparing them for their summative examinations.

*“The registrars help to hone things down to what’s examinable for us. Sometimes the senior consultants - the experts in their fields tend to gloss over the basics, which we don’t even have. Registrars were better teachers because they understood our level, they were extremely approachable”. (S 12)*

Students were most impressed by the **consultants’ history taking ability**, whereas they felt the registrars were less practiced, and still finding their way. Positive role models devoted more time to showing by example, the importance of the **doctor-patient relationship**, and the psychosocial aspect of medicine [7].

*“Our tutor was a consultant and had an excellent manner with patients. He was so calm and so matter-of fact, everyone kind of calmed down around him and he was extraordinarily good with patients. So I would emulate his style. Calm, professional. He gave a lot of time to people. He would make it seem like he had all day for you, even if he didn’t…. like he’s not too important to do that.. He was very humble”. (S53)*

Students found watching consultants interact with patients helped them realise the required **balance between professional and compassionate behaviour**. Students made constant reference to their perceptions, both positive and negative, regarding the level of tutors’ empathy, respect and compassion for their patients. Literature suggests that faculty attitudes should reinforce empathy, compassion and caring [25,26].

*“We had a tutor, and the way she really cared for patients was evident. As a student coming though you’re still figuring out where your lines of comfort are in terms of behaving professionally but still having compassion and watching her was great. She’d sit down with the patient and adjust their robe for them and was very happy to be hands on and helpful – in a role that maybe a lot of doctors would consider not part of their job but I think it’s still part of patient care. She was just really happy to make sure her patient was comfortable and clearly cared a lot about them as a person, not just as a person in a bed that they could use to teach us something”. (S11)*

Students were bothered when consultants displayed a lack of **empathy for patients**. They felt that some consultants had become insensitive to the patients’ needs over time.

*“One thing I’ve seen in certain consultants is a lack of patience for the patients, because sometimes they’re really quick. When you have people who really take the time and talk to the patient and really allow them to express what they want to, instead of cutting them off to get the point, that’s important to us”. (S13)*

Further to this, students expressed a sense of despair at **their inability to advocate for the patient**. A sense of powerlessness has been previously reported by students in regards to patient centred care and values [27].

*“The empathy for the patient, probably foremost is really, really important because there have definitely been some excruciating periods when you’re thinking should we really be doing this exam right now on this patient? We already feel awkward as a student. And a bunch of students together crowding a sick patient in hospital…..our tutor needs to be the leader to understand the situation and be, look, we stop here, and we go discuss it”. (S10)*

*“The patient fell asleep while were there, and he woke her up….They need to understand that the patient is actually doing us a favour by talking with us …and giving them the respect and compassion…there wasn’t a lot of compassion there”. (S24)*

Demonstration of **etiquette and politeness** was important to students.

*“The patients often get what they call student fatigue, where they’ve seen so many students and they’re sick of telling their story over and over. My tutor was always very big on getting permission for the whole group to come in and sit down and chat to the patient, so it seemed like you had a lot of time. We had other ones where they just barged into the room, saying ‘this is my group. We’re all going to do an exam on you now’”. (S15)*

**Respect for the patient**, and the way in which the tutor spoke about the patient was important to the students, whether they were at the bedside or away from it. It has previously been shown that students are disappointed by tutors who display derogatory humour within their teaching [28].

*“When talking about the patients, the specialist or the registrars are often quite flippant – they’re like – well, they’re crazy. I think they can deal with it more tactfully. I hope that when we become doctors we take that with us and realise that if their problem isn’t something that we can organically find, then you don’t necessarily jump to the conclusion that it’s something that’s mental health, and even if it is, to give that a bit more respect”. (S50)*

*“We had a tutor who was humble and so professional. After the tutorial he would find another room to talk to you. He would never talk in the hall about the patient, he was very respectful, and never called them by their pathology or their room number. He always said, ‘we’re going to see Mrs X or Mrs X’. He always called them by their names. He was very respectful and professional. He would never speak ill of a colleague. He was very moral”. (S9)*

Students also conveyed the sentiment that a tutor’s **concern for patients should be genuine**. *“We nicknamed one tutor “Dr Psychopath” because he was extraordinarily charming – in a superficial way. He would talk to patients in a charming way – but there was something dead behind his eyes – it was very clear to us – and maybe to the patients – that he didn’t actually care, and he was just putting on a show. I never want to be like that because it gets you into dangerous territory – you stop caring about someone as a human being”. (S6)*

### Teaching skills

It was important to students that their tutors took the **time to develop a rapport** with them, which has been shown to assist in development of students’ autonomy and competence [29].

*“I like it when they’re involved with the group, when they like spending some time to get to know us, that helps. We had one doctor who by the end, it was like we were talking to a colleague – it facilitated our learning a lot better. He took the time to get to know us”. (S15)*

In particular, a sense of belonging was fostered by tutors when students were treated as **team members**, with similar goals and purpose [30,31].

*“One thing I really liked and appreciated is when they recognize that we will be their colleagues down the road, and there’s obviously a huge knowledge gap and a large age gap, and there’s that hierarchical difference, but there were tutors who treated us as a team, a few different words here and there actually helped – made us realise that what we’re doing is not just playing”. (S20)*

Students were resentful of being made feel they were a **burden to the tutors**.

*“Some consultants just come in and out for the tutorial and we’re seeing they’re really busy and we don’t want to ask them questions and we don’t want to hold them back too late, even though we have a burning question, it looks like they’re rushing everywhere, and we just don’t want to burden them. It affects your relationship and how we perceive and how I will act later as a tutor”. (S8)*

Students want tutors who are more **involved with their academic development and growth** as a student entering the medical profession. They like to be given additional guidance, invited to their tutor’s outpatients, theatre or ward rounds.

*“The tutors who gave us guidance were good. They told us what lectures were good, what book is great. It helps you find your feet. Or tell you what equipment you need for the block – like you need to buy a tendon hammer, those sorts of things”. (S19)*

Students wanted tutors who would provide them with their **time**.

*“The tutors who made me feel like they had the most time in the world probably had the least. We had the Associate Dean of the clinical school, obviously really busy, but never made us feel like we were in the way or annoying or rushed”. (S13)*

*“I had a registrar. She was always 20 minutes late, and then she’d ask what we were doing, quick lets go find a patient, and then she’d rush us in and rush the patient, and you’d learn nothing, and then she’d rush you out and she’d go”. (S16)*

Students valued tutors who created a **positive learning environment.** Students found teaching itself a form of role modelling. There were impressed (or not) by the style of teaching; the structure of teaching; and very importantly, whether or not a tutor had taken the time to prepare. These three factors were key to forming an impression of integrity, and were factors that students would (or would not) wish emulate in their future careers. Students were upset by negative interactions with their tutors.

*“Some tutors teach by interrogation and humiliation. I find it really unhelpful and very upsetting and counterproductive… I like it when I can just try and answer instead of being scared of being wrong”. (S3)*

At the same time, students did want to have some kind of **expectations** placed on them by the tutors.

*“I like to be challenged though. I like it when they push you, but without making you terrified……….. Our tutor would make us do homework, and she knew what we had that was important coming up. She knew what was going on in the curriculum”. (S17)*

Students expected tutors to have a **structure to their teaching**, and be provided with a context. Teachers who are experts in the cognitive and psychosocial aspects of the medical profession are key to role modelling, and making explicit aspects of the profession that may be unspoken, such as the reasoning behind their decision making processes [32,33].

*“I had one tutor who really brought together what we’re studying with the clinical stuff. We’d see a patient and we’d go into a room and talk about it later, their symptoms, how they got the disease, the whole process through to management, which brought it together really well. You remember a lot more that way”. (S44)*

*“This tutor gave us a background and then we did the examination and then we’re allowed to look at their scans and CT and that’s very helpful to putting it all together. And telling me as a student what I would do in the future. I have no idea how the health care system works, but when they go through it, they present at ED, we did the scan, we did this examination, this history, then we looked at the scan and found this result, told us how it all fits in the flow”. (S40)*

Students expected their tutors to provide opportunities for **patient interaction**. Learning is context dependent, and therefore, activities that take place in an authentic environment, such as at the bedside with real patients, is the best place for learning to take place [33]. The teacher, as the role model, is responsible for providing this environment. This can be an arduous task, but it is important.

*“Our GP (general practitioner) tutor rarely took us to the wards. He’d say, ‘unless you have your own patients, I have no idea where the patients are’. So we just stay in our tutorial room, and that’s what it’s continuing to be this year”. (S33)*

Students felt that **consistency** in a communications tutor was beneficial. However, they preferred to experience teaching from consultants from different specialties for the examination tutorials according to the block.

*“I think it would be really good for communications, for that continuity. But for examinations it’s kind of nice to have someone with speciality in that area who can show you what they actually do. It’s nice to get different insights into different medical professions”. (S17)*

Students wanted immediate **feedback** from tutors in order to improve their own performance, but also as a way to model provision of feedback to their own peers. Carefully structured feedback on tasks has been shown to increase student motivation [29].

*“We had a tutor who saw us as developing students, who gave us really good feedback. She gave us constructive feedback, and it is really nice to have someone who’s supportive, but also who can criticise you* and *you can take on the criticisms to improve. It also helps us with critiquing ourselves and critiquing our peers as well”. (S15)*

*“My tutor was really encouraging. He gave us a lot of positive feedback. He was big on saying something positive, something to improve on, then something positive again. He’d always give us one manageable thing to work on that week and then we could build on that, which wasn’t overwhelming”. (S23)*

### Personal qualities

Students expressed their appreciation of seeing how important **interdisciplinary contact** is within the hospital. It has been previously described that negative role modelling may include prevalence of hierarchy and exclusivity by senior specialists (consultants), gender issues, and criticism of peers or departments [34].

*“Our consultant introduced us to the occupational therapist who was talking to the patient. He introduced us in front of the patient, and told us what she does for the patient. This creates that kind of multidisciplinary mindset that we all should really have and that sometimes we don’t get exposed to, so to have that consultant introduce us to that kind of world and that kind of thinking definitely has impacted me”. (S19)*

*“The (consultants’) interactions with nurses, with other consultants and with the students is important to me – our tutor is always courteous, very professional. It’s a good learning experience to know how to communicate with others in the hospital and see the team environment”. (S7)*

Students expect their tutors to **be prepared** and to have an adequate **understanding of the curriculum.**

*“If they were a good person, and they had integrity, and they always tried to help us, we always wanted to do most work for them. Some tutors came very prepared, they clearly had read the handbook, knew what we were supposed to do, knew what they were going to teach us, then if we didn’t know, we’d feel pretty terrible”. (S11)*

*“We had a few tutors who didn’t really know anything about the Sydney Medical Program. Even just one sheet saying where first years were at. Instead of tutors walking into the tutorial and saying ‘so what’s our topic for this week?’ that’s not very encouraging”. (S18)*

Students wanted to see **enthusiasm** from their tutors for their subject, and also passion for teaching.

*“They need to be passionate about what they’re teaching. The ones that stick in my head are the ones that really enjoy their field and want to teach us everything about their field”.*

*“It was clear our tutor loved teaching, and he was willing to take an extra hour out of the day to spend with us to make sure you understood what he had just taught. He went the extra mile to help us learn, beyond his duty. He created little booklets for us of knowledge sets, quick tips and everything”. (S27)*

Positive role modelling can influence the **career choices** made by students, particularly when doctors demonstrate a passion for their work in the clinical setting. Clinical tutors should be aware of their impact on the recruitment and retention of learners into the medical profession [35]. Students expressed concerns about tutors who were negative about their career choice.

*“I had a tutor who was very cynical about medicine as a profession. When you’re in first year that is quite discouraging. For me this was quite a big life decision, and to have someone tell you every week when you rock up to the hospital that you’ve picked a ridiculous career path – it’s pretty discouraging”. (S51)*

## Discussion

In accordance with current literature, our findings illustrate that clinical tutors play an important role in socialising and supporting new students to develop their professional identity on entering a career in medicine [35-38]. Students learn via observation, imitation and modelling their tutors [35]. Our study revealed that role modelling played a critical role in influencing students’ motivations and choices in what behaviour to engage in. Essentially, students perceived that during the clinical tutorials, the tutors were demonstrating their professionalism beliefs and values of the medical profession. Clinical tutors’ demonstrations of *clinical competence*, *teaching ability* and *personal characteristics* were important to students. Students recognised characteristics and behaviours that they want to emulate in the future as medical practitioners, and those that they do not want to emulate. These behaviours have been summarised in Table 1.

**Table 1:** **Positive and negative role modelling characteristics and behaviour identified by medical students**

Behaviour identified by students as positive (they would like to emulate in the future)	Behaviour identified by students as negative (they would not like to emulate in the future)
**Clinical attributes**	
• Good knowledge of general medicine	• Inability to impart knowledge at the student level
• Articulate history taking skills	• Talking about patients without respect
• Ability to explain and demonstrate clinical skills at appropriate student level	• Lack of empathy or compassion patients
• Empathy, respect and genuine compassion for patients	• “Fake” empathy or compassion for patients
**Teaching skills**	
• Development of a rapport with students	• Lack of time for students within and outside of tutorials
• Provision of time towards the growth of students academically and professionally	
	• Poorly structured tutorials
• Provision of a positive learning environment	• Humiliation of students
	• Poor understanding of the curriculum and assessment requirements
• Structured tutorials with clear expectations	
	• Lack of meaningful feedback
• An understanding of the curriculum and assessment requirements	• Lack of patient interactions
• Immediate and meaningful feedback	
• Provision of patient interaction	
**Personal qualities**	
• Respectful interdisciplinary interactions	• Lack of preparation for tutorials
• Preparedness for tutorials	• Lack of enthusiasm for teaching
• Punctuality	• Negative regard for the medical profession
• Enthusiasm for teaching and the subject	
• Demonstration of a passion for their career choice	

### Clinical attributes

Although students were impressed by senior specialists’ (consultants’) ease and skill in their demonstration of clinical skills at the bedside, the students’ concern for empathy and respect shown to the patient appeared paramount. Doctors who appeared insensitive to the needs of patients were perceived as negative role models [39]. As shown in previous studies, we found that within the hidden curriculum, there can be negative influences on the clinical learning environment. These include undesirable behaviours by tutors, such as tutor-centred patient interactions in order to save time; the humiliation of students as a way to encourage learning; and negative remarks about colleagues [40,41].

### Teaching skills

Interactions with teachers have the greatest impact when the learner has *adequate time* within tutorials [33]. Certainly, this was a recurrent theme within our student feedback. When tutors did not dedicate enough time to students both within and outside of tutorials, students felt they were a burden to the tutors, which prevented them from asking questions and fully engaging in learning activities. It has also been suggested that teachers have the greatest impact when there is *continuity,* giving the students the opportunity to see patterns of behaviour and multiple roles that are relevant to the medical professional [32,33]. While we found that continuity was important to our students within their communication tutorials, they enjoyed engaging with a mix of specialists during their physical examination tutorials. Students found that where continuity did exist, they were able to build a rapport and relationship with their tutors that enhanced their learning. Tutors were able to highlight the positive aspects of their performance, building on strengths, and providing immediate feedback where corrections were needed [41]. Students reported that once they became comfortable with their tutors, they were more likely to ask questions freely [41,42]. Previous studies have found that that appropriate supervision and support can be provided when tutors develop an in-depth knowledge of students’ ongoing development and devise appropriate challenges [42].

### Personal attributes

The actions of tutors are important in shaping the culture of the learning environment, and helping create a relaxed, collegial atmosphere [33]. In line with previously reported positive examples of role modelling, our students appreciated their tutor’s respectful interactions with other health professionals [43]. They felt that these interactions helped with the development of their own professional identity, making them feel part of an extended professional community [43]. Additionally, students reported that integrity was displayed by tutors when they prepared for their tutorials, were punctual, and displayed an understanding of the curriculum and assessment requirements through their teaching. Students expected to see both a passion for teaching, and a passion for the topic. Role models have been shown to have a positive impact on student career choices when they engage and involve students in their clinical setting and through demonstration of a passion for the work within their medical field [44,45]. Our students found it discouraging when their tutors were negative about medicine as a career choice, potentially adversely influencing student motivation [34].

Given that the teaching of professionalism is highly content dependent, positive role modelling remains a very important method of transmitting medical professionalism in learners [20]. Being cognisant of students’ perceptions of the attributes of outstanding role models in medicine may help medical educators to develop strategies to attract and retain the best clinical tutors. Many of these attributes associated with excellent role models relate to skills that are learnt skills [7]. By reflecting and developing these attributes, clinical tutors can improve their impact on students as role models [46]. This has important implications for medical schools in terms of developing their clinical tutors. A crucial step in enhancing role modelling is for clinical tutors to assume a conscious awareness of role modelling in all clinical environments [6], and for faculty to help them to do so. Additionally, consideration should be given to encouragement by faculty to critically assess the attributes of their clinical tutors, with the intention to only select those displaying attributes that are perceived as ethical and useful [41].

### Limitations

The limitations of this study include the small sample size, and the fact that a convenience sample was used, and the sole qualitative nature of the study.

## Conclusion

Excellence in role modelling entails demonstration of excellent clinical care, teaching skills and personal characteristics [4-6]. Our findings reinforce the important function of clinical tutors as role models. Our students perceived key positive examples set by their clinical tutors to include a good knowledge of general medicine; empathy and respect for patients; a positive learning environment; understanding of the curriculum; meaningful feedback; and enthusiasm for both teaching and medicine. In effect, the clinical tutors act as socialising agents in demonstrating the expected culture and professional values of both medicine as a profession and the hospital as an organisation [47], which has implications for faculty development and recruitment.
